# A Review of the Emerging Role of Silk for the Treatment of the Eye

**DOI:** 10.1007/s11095-018-2534-y

**Published:** 2018-11-05

**Authors:** Simon H. Tran, Clive G. Wilson, F. Philipp Seib

**Affiliations:** 1 37D Biosystems, Inc., 2372 Morse Avenue, Suite 433, Irvine, California 92614 USA; 2 Strathclyde Institute of Pharmacy and Biomedical Sciences, University of Strathclyde, 161 Cathedral Street, Glasgow, G4 0RE UK; 3 Max Bergmann Center of Biomaterials Dresden, Leibniz Institute of Polymer Research Dresden, Hohe Strasse 6, 01069 Dresden, Germany

**Keywords:** *Bombyx mori*, drug delivery, eye, ocular, silk fibroin, tissue engineering

## Abstract

Silk is a remarkable biopolymer with a long history of medical use. Silk fabrications have a robust track record for load-bearing applications, including surgical threads and meshes, which are clinically approved for use in humans. The progression of top-down and bottom-up engineering approaches using silk as the basis of a drug delivery or cell-loaded matrix helped to re-ignite interest in this ancient material. This review comprehensively summarises the current applications of silk for tissue engineering and drug delivery, with specific reference to the eye. Additionally, the review also covers emerging trends for the use of silk as a biologically active biopolymer for the treatment of eye disorders. The review concludes with future capabilities of silk to contribute to advanced, electronically-enhanced ocular drug delivery concepts.

## Introduction

**Fig. 1:**
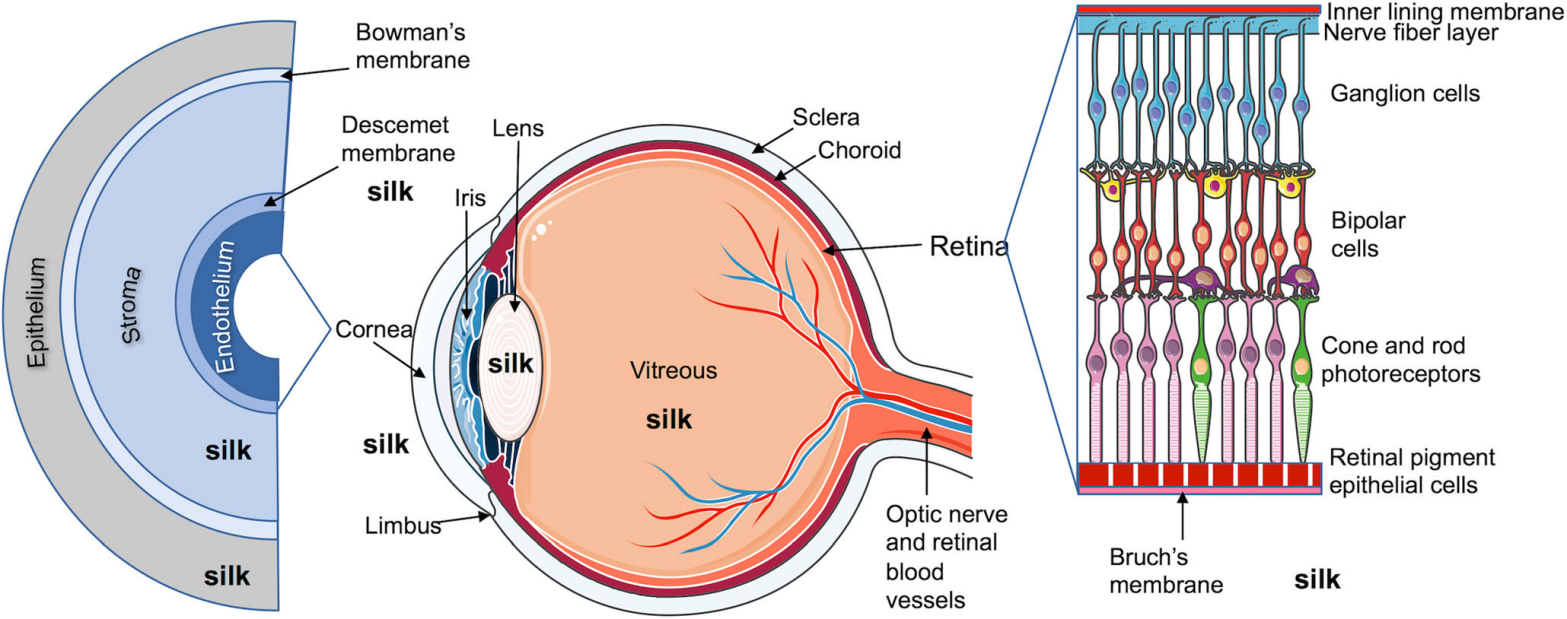
Diagram of a human eye showing expanded corneal layers and retinal layers. Annotated silk fibroin figures indicate the ocular spaces where silk was studied. Figure adapted from Servier Medical Art.

Humans have appreciated silk cloth for millennia for its lustrous texture and remarkable physical properties (1). The ability to unravel the thread from the silk cocoon and weave fabrics with the cleaned thread has been truly transformative (1). Repurposing the silk fibre for medical applications changed the medical landscape (1) ultimately resulting in sterile silk sutures still in use today (2), especially for delicate procedures like eye surgery. Our ability to apply top-down and bottom-up approaches to generate silk with the required properties has resulted in an explosion of potential applications (1,3,4). In particular, our ability to fully regenerate the silk cocoon into an aqueous silk solution and to use genetic engineering to produce recombinant silks with molecularly defined composition. These new additions to the silk toolbox now allow the fine-tuning of silk function by manipulation of silk across all scales, including primary sequence, chemical functionality, and secondary and tertiary structure, as well as formatting it from the nano- to macroscopic scales (1). Recent reviews detail the current and emerging trends in the use of (recombinant) silk for biomedical (1,3) and advanced manufacturing (4). This review now describes the use of silk for the treatment of eye disorders by closely examining the use of silk for eye-related tissue engineering applications, as well as for drug delivery to the eye (Fig. 1).

The eye is enclosed within the skull and anchored by muscles and tendons; therefore, the spaces around the eye provide convenient sites that could serve as drug depots for materials such as silk-derived hydrogels (provided that vision is not impaired and the formulation does not physically obstruct eyeball rotation). The material could be moved under the orbital fascia, such as the conjunctiva, and placed in the space between the globe and the skull (a subtenon injection) or within the eyeball. The administration of subconjunctival injections by nursing staff is a common practice in the clinic (5); for example, sustained-release glaucoma medicine can be administered by subconjunctival injection. Therefore, acceptance by patients is likely to be high (6). Canavan and colleagues provide a useful review of the application of this subtenon technique in anaesthesia (7). Volumes of around 2 mL can typically be introduced without problems. Creating depots more posteriorly might be more desirable to avoid clearance mechanisms, but this reduces access and could compromise the vascular system. Retrobulbar injection (i.e. injection behind the eyeball) has also been practiced for many years, although it is not without risk (8,9). Awareness of the risk has been raised by experience with three-dimensional remoulding of facial tissue conducted by cosmetic surgeons, who have reported that autologous fat injection can cause irreversible blindness due to embolic occlusion of arteries. The alternative is to inject biomaterials such as hyaluronate, which can be degraded quickly in an emergency. For example, Carruthurs and colleagues have illustrated that retrobulbar injection of high-dose hyaluronidase can prevent blindness if carried out in the short critical window before irreversible retinal hypoxia occurs (10) following the use of hyaluronic acid based fillers. Such a safety consideration to remove a matrix safely in an emergency is an unexpected and valuable attribute. Finally, the potential space is surprisingly large: a complete peribulbar injection, filling all the space including the intraconal fat space can accomodate larger volumes - typically 6 to 12 mL.

Most studies to date that have investigated the use of silk for ocular drug delivery and tissue engineering applications have used everyday silkworm silk (i.e. *Bombyx mori* silk) (Table I). For this reason, we first provide a brief background of the *B. mori* silk structure (reviewed in more detail in (28)).

**Table I:** Use of Silk for the Treatment of the Eye

Author(s) and year	Silk processing: type; degumming time; post casting treatment; final format	Ocular treatment type	Biological assessment
Wu *et al*. (11)	*B. mori*; 30 min; water-annealed at 25°C 20 mmHg 2 h; autoclaved 121°C 20 min; patterned silk film	Stroma replacement; substrate for human corneal stroma stem cells and corneal fibroblasts	*In vitro;* stroma stem cells *vs* fibroblasts on patterned silk films; tested cell morphology, gene expression, protein expression, and extracellular matrix expression and structure.
Liu *et al*. (12)	*B. mori*; 60 min; water-annealed 10 psi 4 h; sterilized 160°C 2 h; silk Film	Epithelium replacement; substrate for human and rabbit corneal limbal epithelial cells	*In vitro*; silk *vs* amniotic membrane; tested cell morphology, differentiation, stratification.
Lawrence *et al*. (13)	*B. mori*; 40 min; water-annealed 10 psi 4 h; sterilized 70% EtOH; patterned silk film	Epithelium replacement; substrate for human corneal limbal epithelial cells	*In vitro*; flat *vs* patterned silk film *vs* glass; tested cell morphology, density, cytoskeletal structure.
Biazar *et al*. (14)	*B. mori*; 30 min; electrospun; dried in vacuum 2 days at 25°C; Nanofibrous Silk Mat	Epithelium replacement; substrate for human limbal stem cells	*In vitro*; random- *vs* oriented-silk mat *vs* amniotic membrane; tested cell viability, karyotype, cellular markers
Suzuki *et al*. (15)	*B. mori;* 60 min; PEG-treated; water-soaked for 3 days; PEG-treated silk film	Epithelium replacement; substrate for human corneal epithelial and limbal cells	*In vitro*; silk film *vs* PEG-treated silk film; tested film strength, permeability, cell adhesion and proliferation
Kang *et al*. (16)	*B. mori;* 40 min; Water-annealed 10 psi 4 h; patterned silk film	Epithelium replacement; substrate for human corneal epithelial and limbal cells	*In vitro*; flat *vs* patterned silk film; tested cell alignment morphology, adhesion, cellular markers, cytoskeletal structure.
Li *et al*. (17)	*B. mori;* 60 min; PEG-treated; sterilized 75% EtOH; silk film	Epithelium replacement; substrate for rabbit limbal epithelial stem cells	*In vivo*, rabbits; limbal stem cell deficiency; tested corneal thickness, neovascularization, clarity; contralateral eye as control; procedure-associated adverse events, not specific to silk.
Wang *et al*. (18)	*B. mori;* 30 min; water-annealed −25 mmHg 2.5 h; sterilized 70% EtOH; porous and patterned Silk films and sponges	Stroma replacement; substrate for human corneal epithelial and stromal stem cells and chicken dorsal root ganglion	*In vitro*; co-cultures *vs* single culture; liquid *vs* air-liquid interface; tested density and length of neurons, cell proliferation, and gene expression.
Shadforth *et al*. (19)	*B. mori;* 60 min; water-annealed −80 kPa 6 h at 25°C; silk film	Bruch’s membrane replacement; substrate for retinal pigment epithelial cells	*In vitro*; silk *vs* polyester membranes; tested cell morphology, gene expression, trans-epithelial resistance, phagocytosis and growth factor secretion function.
Vazquez *et al*. (20)	*B. mori*; 30 min; water-annealed under vacuum 24 h; sterilized 70% EtOH; silk film	Descemet membrane replacement; substrate for human and rabbit corneal endothelial cells	*In vitro* and *in vivo*, rabbits; tested transparency, gene expression, cell growth, integration and proliferation; no signs of immune rejection.
Applegate *et al*. (21)	*B. mori*; undisclosed; crosslinked with riboflavin; elastic hydrogel	Prosthesis as lens	*In vitro*; tested crosslinking mechanism, transparency, adhesion to corneal collagen, film thickness.
Min *et al*. (22)	*B. mori*; 30 min; erosslinked with stilbene; Elastic hydrogel	Prosthesis as photonic crystals	*In vitro*; tested optical response in different media and mechanical stresses.
Abdel-Naby *et al*. (23)	*B. mori;* 45 min; autoclaved (15 psi, 121°C, 30 min); silk solution	Wound healing on ocular surface (eye drops)	*In vitro*; soluble silk solutions *vs* placebo PBS; tested cell migration rate, cell viability, cell adhesion, and cell proliferation.
Abdel-Naby *et al*. (24)	*B. mori;* 45 min; autoclaved (15 psi, 121°C, 30 min); silk solution	Wound healing on ocular surface (eye drops)	*In vivo*, rabbits; soluble silk solutions *vs* placebo PBS; tested silk stability, cell proliferation, cell adhesion, cell markers Ki-67 and MMP-9. No adverse events were reported.
Kim *et al*. (25)	*B. mori;* 60 min; silk solution	Dry eye (eye drops)	*In vivo*, mice; soluble silk solutions *vs* placebo PBS; tested tear production, corneal irregularity, epithelial cell detachment, goblet cells density, inflammatory factors TNF-α, MMP-2, MMP-9, ICAM-1, and VCAM-1. No adverse events were reported.
Dong *et al*. (26)	*B. mori;* 40 min; degradation by CaCl_2_; silk-coated liposomes solution	Topical drug delivery (eye drops); mucoadhesive polymer for ibuprofen-loaded liposomes	*In vitro*; silk-coated liposomes *vs* liposomes; tested morphology, particle size, zeta potential, encapsulation efficiency, *in vitro* drug release, cell cytotoxicity, corneal permeation, and cell adhesion.
Lovett *et al*. (27)	*B. mori;* undisclosed; self-assembled hydrogel	Age-related macular degeneration; intravitreal drug delivery for bevacizumab in hydrogel format	*In vitro* and *in vivo*, rabbits; bevacizumab-loaded silk hydrogel *vs* bevacizumab solution; tested *in vitro* release, *in vivo* pharmacokinetics. No specific adverse reactions to the silk fibroin formulations. Observations associated with intravitreal injections include mild ocular irritation, slight discoloration (redness) and discharge.

### Silk Structure

The domesticated *B. mori* silkworms spin their cocoons from 2 main proteins: silk fibroin and sericin. The silk fibroin protein is composed of several subunits—the heavy chain, the light chain and glycoprotein P25 (29)—whereas sericin is a single hydrophilic protein having a molecular weight ranging from 20 to 400 kDa (30).

The silk fibroin heavy chain is the largest of all the fibroin subunits, with a molecular weight of approximately 350 kDa (29). The mechanical strength of the silk fibre is imparted by the 12 crystalline regions of the silk fibroin heavy chain (31). These crystalline regions, embedded within an amorphous matrix, are composed of highly structured β-sheets. The most abundant silk sequence contributing to these β-sheets is a highly repetitive GAGAGS motif (31,32). The light chain of the silk fibroin, approximately 25 kDa in size, has no repeating amino acid sequence and is covalently attached to the heavy chain by a single disulphide bond at the C-terminal (33). Although glycoprotein P25 is about the same size as the light chain (27 kDa) (29), it is not covalently connected to the heavy chain; instead, it associates with the heavy–light chain complex through hydrophobic interactions (34).

The silk fibroin is assembled from various secondary structures, including β-sheets, α-helices and turns. Consequently, its conformation can be impacted by mechanical stresses (i.e. shear, strain), solution chemistry (i.e. solvents, pH, ions) and temperature (35). Three conformations—silk I, silk II and silk III—are recognised for silk fibroin. Silk I consists mostly of α-helices, whereas the majority of silk II is composed of β-sheets in the secondary structure. The aqueous solubility is much higher for native silk I, at approximately 26–30 wt.% (36,37), than for regenerated silk (detailed below), which typically aggregates at these high concentrations. The high solubility of the silk I conformation enables the silk fibroin to exist as a concentrated solution in the silk gland, where it awaits spinning into silk II (36). The structure of silk III is less well understood; however, at an air-water interface, silk III contains helical structures that function similarly to a surfactant, separating hydrophilic serine residues from hydrophobic alanine residues at the opposite sides of the interface (38).

The conformation of the silk fibroin can directly impact the mechanical strength of the resulting silk fibre. For example, Wang and colleagues showed that ions, such as Na^+^, K^+^, Ca^2+^ and Cu^2+^, can alter the conformation of the silk fibroin, thereby affecting the fibre strength (39,40). The silk thread is bifilar because each silk filament is derived simultaneously from the silkworm’s paired silk glands. Within each gland, a drop in pH triggers silk self-assembly, which drives the transition of liquid silk into its fibre format. The two silk filaments are combined and coated with the glue-like sericin protein (41), before the silk thread is pulled (i.e. ‘pulltrusion’) (42) from the spinneret. This inherent self-assembly of silk fibroin, triggered by the aforementioned environment factors (i.e. pH, ions, etc.), has been widely exploited to develop novel silk formats using the regenerated silk fibroin (detailed below); sericin is removed during the silk fibroin regeneration process.

### Sericin – Friend or Foe?

The raw bifilar silk thread contains sericin, which encases and physically combines the two silk fibroin filaments. Sericin is a protein with a large number of neutral polar functional groups. It contributes between 25 and 30% of the cocoon weight and forms the gummy layer that holds the cocoon together. Sericin is classified according to its solubility in water into 3 different categories: sericin A, B and C (from most soluble to least soluble in aqueous solution) (30). Sericin is typically removed during raw silk processing, and yielding about 50,000 tons of sericin in 2002 (43), a figure that is still quoted as approximate world tonnage in 2017. Small sericin peptides are soluble in cold-water and are recovered at the early stages of raw silk processing, whereas the larger sericin peptides are obtained during hot water degumming of the silk. As a waste product of the extraction process, sericin is used as an inexpensive filler for other polymers, including polyurethane foam, films and foams that include polyols, catalysts and blowing agents, where needed.

The toxicity of sericin is often debated, and the diversity of data suggests that different preparations may yield different fractions, thereby explaining the observed discrepancies. Historically, sericin has been attributed to adverse reactions in humans reported for virgin silk (reviewed in (44)); however, emerging evidence indicates that sericin is a useful biopolymer with a favourable biocompatibility. For example, sericin hydrogels showed a low *in vivo* allergenic and immunogenic profile, with a response similar to that observed for silk fibroin or alginate (45). Aramwit and colleagues (46) described sericin fractions obtained by different methods, namely heat, acid, alkali and urea extraction, and confirmed that the starting material is a mixture of at least fifteen different polypeptide chains (47). These authors examined the zeta potential and particle size of products produced by these different techniques and determined that the urea-extracted fraction was water soluble, whereas the fractions produced by other methods were probably larger hydrocolloids (48). Examination of the viability of an L929 cell line exposed to different extracts at concentrations up to 1 mg/mL showed that the urea-extracted fraction had the greatest toxicity, whereas the other fractions were well tolerated by the cells at concentrations up to 100 μg/mL (48).

The mulberry leaf (the exclusive diet of *B. mori* silkworms) contains many flavonoid antioxidants that become incorporated into the spun silk and especially into the sericin fraction. The biological role of these flavonoids may be to provide chemical protection for the developing moth against pests while the cocoon provides physical protection. In small rodents, these “contaminated” sericin materials show pharmacological effects as diverse as improved stamina and increased intestinal absorption of metals (49). Tissue regenerative effects have also been suggested, and the potential of nanofibrous mats based on silk sericin/PVA electrospun material has been explored following subcutaneous implantation (50). Data in mice indicated good biocompatibility and low infection, and the authors suggested that the mulberry leave derived antioxidants within the material might impart superior properties to those of ‘inert’ dressings. Addition of a purified sericin fraction to an immortalised corneal cell culture and to an experimental wound in rats (51) increased the wound healing rate and this response was suppressed by addition of an ERK inhibitor, suggesting that sericin promoted phosphorylation of ERK1/2. However, full wound healing occurred at approximately the same rate as was observed in the controls. Sericin appeared to increase the movement of cells, prompting the authors to look for other cytokine signals that might be affected. These putative medical effects of sericin have inspired many other studies with a view to adding sericin to the human diet. Sericin has also been heavily exploited as an additive in the cosmetics industry. These diverse benefits might aid in overcoming the long-standing and serious problem of environmental pollution associated with sericin generation by the silk industry, thereby encouraging further development of uses for this ‘waste product’ (49).

### Biocompatibility and Biodegradation of Regenerated Silk Fibroin

The mechanical strength of silk makes it an attractive polymer for biomedical applications, such as suturing and tissue engineering. Silk sutures (44), silk garments to treat dermatological conditions (1), and silk surgical meshes (SERI® Surgical Scaffold) (52) are currently the only clinically approved silk products in Europe and North America (1); these products utilise the *in vivo* spun *B. mori* fibre. However, most studies reporting the pre-clinical development of silks for ocular applications utilise regenerated *B. mori* silk (53) (Table I). Here, the silk cocoon is processed into aqueous silk fibroin stock (with similar properties to the native silk stored in the silkworm silk glands) (54). First, the silk cocoons are cut into small pieces and then typically boiled in an alkaline solution (e.g. sodium carbonate) to remove the sericin (53). The resulting silk fibroin is then dried and the higher-order silk structures are disassembled with a chaotropic salt (e.g. lithium bromide, guanidine hydrochloride, urea or calcium chloride). The silk fibroin solution is dialysed against water to remove the salt, ultimately yielding an aqueous silk solution (53). Differences in the processing parameters (e.g. degumming time) can result in regenerated silk fibroins that vary in molecular weight. This, in turn, influences silk fibroin performance, including mechanical strength (55–57).

The regenerated silk fibroin solution typically serves as the starting material for generating novel silk formats, including thin films (58). In the eye, the use of silk films is most feasible in the area of corneal grafting, where the replacement tissue layer of the diseased cornea is often cultured ex vivo and then transplanted back into the patient (59). As a substrate, silk provides a sturdy platform for proliferation of the respective (stem) cells and their differentiation into the matured tissue layer required for transplantation.

The biocompatibility and biodegradation of the regenerated silk fibroin has been evaluated both *in vitro* and *in vivo* often with superior performance than fully synthetic or other biopolymer materials (reviewed in (1,60,61)). Silk is not of mammalian origin (like other clinically used biomaterials e.g. alginate, chitosan etc.); therefore, foreign body responses to silk-based implants and sutures might be expected. Various studies performed in rodents and large animals to evaluate silk fibroin biocompatibility across many different formats, including meshes (52), thin films (19,20), hydrogels (27,45,62), nanoparticles (63–65) and liquid solutions (23,25), have confirmed that silk fibroin induces no significant adverse responses, thereby substantiating the claims that silk fibroin is a biocompatible biopolymer. More importantly, these observations translate well to humans (1). Of course, universal biocompatibility does not exist, so dedicated biocompatibility studies are still required when exploring novel silk applications. Similar considerations apply to the biodegradation of silk fibroin, because the material format, processing conditions and implantation site are expected to impact its degradation profile.

### Silk Sutures

One silk application that dates back several millennia is its use as suture material (1). However, a number of adverse reactions have been reported for virgin silk sutures (66) (reviewed in (44)). Furthermore, braided silk sutures could potentially carry the risk of infection, which is thought to arise due to capillary movement of fluids and the establishment of a biofilm around the silk protein fibres. This potential risk of infection can be mitigated with the application of appropriate coatings, such as the use of an additional levofloxacin layer. For example, Chen and colleagues examined the performance of antibacterial loaded silk sutures in which the silk fibroin strands were dipped in levofloxacin in a polycaprolactone formula to control levofloxacin release (67). Bacterial inhibition zones against *E. coli* showed complete kill after a 24 h incubation. Today, synthetic materials, such as Vicryl®, a copolymer made from 90% glycolide and 10% L-lactide, predominate the suture applications. Nevertheless, the characteristics of silk sutures, such as knot security and lying flat on the tissue surface, remain advantageous. For these reasons, silk sutures are still used today for specialist eye surgery (1).

### Silk for Ocular Tissue Engineering: Corneal Epithelium

Corneal disease is the most common cause of blindness, affecting approximately 27.9 million individuals worldwide (68). Currently, corneal transplantation remains the only acceptable treatment to restore visual acuity (59); however, the increasing demand for and limited supplies of donor corneas from the Eye Bank have created long waiting lists for corneal transplantation in most developing countries (69). The shortage of human corneas has led to some major developments in the field of corneal tissue engineering, particularly in the use of silk films as substrates to grow corneal cells for ocular surface reconstruction (70) at any of the 5 layers of the cornea (from outermost to innermost: the epithelium, the Bowman’s layer, the stroma, the Descemet’s membrane and the endothelium) (Fig. 1).

Presently, the preferred surgical membrane for the corneal epithelium reconstruction is the human amniotic membrane due to its abilities to inhibit inflammation, tissue scarring and angiogenesis. However, the use of this membrane has some drawbacks. Firstly, when compared to other synthetic membranes, such as the silk fibroin, the amniotic membrane has poor mechanical strength, which creates difficulties in handling. Secondly, the amniotic membrane is semi-transparent, which can adversely affect light transmission, an important attribute of the corneal surface. Finally, disease transmission (e.g. HIV, hepatitis, etc.) is a potential risk because the human amniotic membrane comes from human donors. The use of silk fibroin can circumvent these potential issues as demonstrated in preclinical studies by Liu and colleagues using the human amniotic membrane as the control (12).

Liu *et al*. prepared silk fibroin membranes for epithelial cell sheet generation using *B. mori* silk fibroin solution casting, followed by water annealing of the silk film to induce beta-sheets. The resulting silk fibroin film had a 14 mm diameter and 40 μm thickness and was a highly transparent film that could hold its shape after handling. By contrast, the human amniotic membrane prepared in parallel was semi-transparent and prone to folding when handled with forceps. Epithelial cells sheet for subsequent transplantation were generated by seeding human immortalised corneal epithelial cells and rabbit primary corneal epithelial cells on both the silk fibroin membrane and the human amniotic membrane. Cells seeded on the amniotic membrane differentiated faster to give a 3-fold higher density after 72 h of cultivation when compared to the cells seeded on the silk fibroin membrane. However, after 144 h, the cells seeded on the silk fibroin membrane achieved the same density as the cells cultured on the amniotic membrane. The corneal epithelial cells expressed equivalent amounts of keratin 3 and P63a proteins irrespective of their culture substrates (12).

The observed higher proliferation rate in cells grown on human amniotic membrane could be attributed to a more favourable cell adhesion, which is influenced by surface topography as well as the presentation of the RGD peptide (an arginine-glycine-aspartic amino acid sequence required for integrin-mediated cell adhesion) among other, yet defined, factors. Topographic features on the silk surface, specifically the groove pattern, have been demonstrated to assist in the orientation of human corneal stromal stem cells and human corneal fibroblasts during generation of a corneal-like stromal construct (58,71). The orientation of the stromal cells is necessary for conferring transparency of the cornea while the coupling of RGD peptide to a silk film surface is necessary to allow the formation of a confluent layer (11).

Similar to corneal stromal cells, corneal epithelial cells also appear to respond differently to different topographic features of silk film surfaces. Cells grown on silk film surfaces with a parallel line pattern displayed more than 2 fold increase in focal adhesion localisation than on surfaces with a concentric ring pattern or on glass controls (13). The study, however, also found that the presence of a surface pattern affected cell morphology, cell alignment and the cytoskeleton more than just cell proliferation.

In a separate study, human corneal epithelial cells were also found to respond distinctly to surfaces with different pitch and width dimensions (16). Similar to the findings from Lawrence *et al*. (13), the authors found that cell morphology and alignment were most likely to be affected by surface topography. For instance, a nanoscale topography cued the cells to align perpendicular to the patterned edge, whereas larger microscale topography cued the cells to align parallel to the patterned edge. Cell lengths were also 2-fold longer in cells growing on patterned silk films than on a flat silk film surface. The authors also noted that although the overall change in gene expression was similar for all cells grown on surfaces with different topography, the degree of change in gene expression was greater in cells growing on the silk film with nanoscale topography, and those cells also expressed higher levels of genes involved in the development of the cytoskeleton (i.e. paxillin, integrin β1 and vinculin), which is important for focal adhesion.

Biazar and colleagues performed an *in vitro* study that directly compared the utility of silk fibroin *versus* human amniotic membranes for growing human limbal epithelial stem cells (14). The aim of the study was to assess whether human limbal stem cells would retain their limbal stem cell characteristics after being seeded onto either oriented nanofibrous silk mats, random nanofibrous silk mats, or human amniotic membranes. The human amniotic membrane served as a positive control because cells grown on this particular membrane differentiate and stratify into the appropriate intended corneal layer (72).

Several biomarkers (i.e. keratin 3 and 12, nuclear protein P63 and ATP-binding cassette sub-family G member 2) were monitored to assess the cell types. Keratin 3 and 12 marker genes are indicators of differentiated epithelial cells (73), whereas nuclear protein P63 and ATP-binding cassette sub-family G member 2 genes are indicators of stem cells (74). After 15 days of *in vitro* cultivation, cells from all membranes showed higher expression of nuclear protein P63 and ATP-binding cassette sub-family G member 2 genes relative to keratin 3 and 12 expression, suggesting that the cells maintained their “non-differentiation” stage after growing on the membranes for 15 days. However, similar to the findings of Liu and colleagues (12), cell growth and viability were better for cells growing on the amniotic membranes than on silk films. These findings validate the human amniotic membranes as the clinical standard and suggest that second generation silk constructs are required to support the growth of corneal limbal epithelial cells.

In addition to retaining their non-differentiated stem cell stage, corneal limbal epithelial stem cells, when grown on silk film substrates, were also able to differentiate into normal corneal epithelium in a rabbit model of limbal stem cell deficiency (17). Typically, patients with limbal stem cell deficiency exhibit increased neovascularisation and decreased corneal clarity (75). These authors successfully replicated the limbal stem cell deficiency conditions with their rabbit model (17). The surface roughness and tensile strength of the final silk films were improved by mixing and subsequent removal of PEG 400 from the silk fibroin solution. The authors asserted that this additional PEG 400 treatment was essential to provide a silk fibroin film with sufficient strength for surgical manipulation (17). However, in a separate study, Suzuki and colleagues reported no improvement in the tensile strength of the resulting silk film following PEG treatment (15). These conflicting data could be attributed to differences in the processing parameters. For example, Suzuki and colleagues generated samples with a high PEG content (i.e. 1 part 100% PEG_300_ to 2 parts 1.78% silk fibroin (15)) whereas Li and colleagues (17) used substantially less PEG (i.e. 1 part 40% PEG_400_ to 100 parts 4% silk fibroin). Li and colleagues showed that rabbit limbal epithelial stem cells responded best to tissue explant and single cell-suspension cultivation methods rather than a cell cluster cultivation method (17). Cells harvested from tissue explants and single cell-suspension cultures expressed higher levels of nuclear protein p63α and the protein ATP-binding cassette, sub-family B, member 5 (i.e. limbal stem cell markers) than did cells harvested from cell cluster culture. In the limbal stem cell deficiency rabbit model, the authors showed that the limbal epithelial stem cell/silk fibroin grafts inhibited the formation of new blood vessels and restored the corneal epithelium, providing evidence that cultured limbal epithelial stem cells can differentiate into normal corneal epithelium to replace a diseased epithelium (17).

### The Third Dimension

Silk fibroin can be utilised as a support material and the growth and expression of important messenger molecules can be measured; however, the removal of cells from the extracellular matrix and monolayer culture during harvesting can increase apoptosis and the rates of cell death. This phenomenon has been extensively studied during the collection and transport of pancreatic islet cells from human donors (76). Three dimensional constructs, by contrast, can serve as artificial niches; for example, silk presenting the RGD motif assists in cluster formation and mimics the extracellular matrix (77). The RGD sequence can be grafted onto *B. mori* silk using standard carbodiimide chemistry (78) (because *B. mori* lacks the RGD motif) or, as shown by the Hedhammar group using recombinant silks, the sequence can be cloned into the repetitive part of spider silk 4REpCT protein (79). The pancreatic islet explant work by Hedhammar and colleagues showed that it is plausible to transplant insulin secreting islet-like clusters from mouse and human primary cells grown on a silk foam into the mouse eye. The transplanted clusters remained capable of releasing insulin (80) and were vascularised and innervated within the eye (81).

### Silk for Ocular Tissue Engineering: Corneal Stroma

The stroma comprises about 90% of the cornea thickness and consists mostly of collagens, proteoglycans and cells (82). It is a complex layer, with highly ordered microstructures that interact with the epithelium layer and nerve cells. Diseases associated with impaired corneal innervation include corneal ulcers, which are marked by decreased corneal sensitivity and alterations in the corneal epithelium, nerves, keratocytes and endothelium (83,84). A recent study by Wang and colleagues focused on the development of a silk corneal scaffold that included the epithelium and the stroma, along with innervation (18). The authors used human corneal stromal stem cells, human corneal epithelial cells and chicken dorsal root ganglion; the latter can interact with human cells and mimic corneal innervation (85,86). Films were generated by solution casting of silk fibroin spiked with PEG onto a polydimethylsiloxane template (either patterned or plain), followed by drying and water annealing (18). The PEG was then leached from the samples to generate porous silk films that would ultimately allow mass transport. The films either contained patterns or were biofunctionalised by stamping (with keratinocyte growth factor, hepatic growth factor, epithelium growth factor and nerve growth factor). Patterned silk films were seeded with human corneal stromal stem cells, while human corneal epithelial cells were seeded onto the porous silk films. The silk sponge was seeded with chicken dorsal root ganglion. The three dimensional corneal tissue model was assembled by stacking the cell-seeded patterned and plain silk films; these were then surrounded with the porous silk sponge to provide innervation. These three-dimensional co-cultures were cultivated in either liquid or an air-liquid interface and were then compared with single cultures (18).

After 28 days of cultivation, the resulting engineered corneal tissue achieved a transparency comparable to that of a porcine cornea. Due to the simulation of the microenvironment within the stroma, cultivation in an air-liquid interface was deemed more effective than the liquid interface and attained about a 2-fold increase in axon density. Furthermore, cultivation of the co-cultures in liquid led to a decrease in numbers of human corneal stromal stem cells when human corneal epithelial cells were included in the system. This result was not observed when the co-cultures were cultivated in the air-liquid interface. These findings indicated that human corneal epithelial cells are more sensitive to the culture conditions than are human corneal stromal stem cells. Therefore, the development of a successful scaffold should preferably incorporate cultivation in an air-liquid interface as this mimics the natural cornea microenvironment.

Chicken dorsal root ganglion was used to innervate the synthetic silk fibroin scaffold and innervation was observed in all co-cultures (18). The complex interactions of different corneal cells and their extracellular matrix within this silk fibroin scaffold showed its potential usefulness as a stroma alternative for the study of drug development, disease intervention and general corneal physiology.

### Silk for Ocular Tissue Engineering: Corneal Descemet Membrane

Ocular diseases involving the corneal endothelium have often been addressed by surgical techniques such as Descemet membrane endothelial keratoplasty and Descemet stripping automated endothelial keratoplasty (reviewed in (87)). These procedures involve the transplantation of the patient’s Descemet membrane, along with the endothelium, with the donor’s graft. Tissue engineering using silk fibroin could potentially supplant the need for donor grafts. Similar to corneal epithelial cells and stromal cells, the corneal endothelial cells can be cultured on silk fibroin substrates, which act like the Descemet membrane, as shown for the artificial endothelial graft constructed by Vazquez and colleagues (20). They cast the silk film from a 5% *w*/*v* silk fibroin solution and obtained a thin film with a thickness of 10 μm. The final silk fibroin film was water-annealed.

Both human and rabbit corneal endothelial cells have been successfully cultured on silk fibroin films to form silk fibroin endothelial grafts (20). These endothelial grafts containing rabbit corneal endothelial cells were successfully transplanted into New Zealand white rabbits in a Descemet membrane endothelial keratoplasty procedure. The rabbit silk fibroin endothelial grafts integrated fully with the corneal tissue and showed a similar thickness and cell count as observed in the contralateral cornea. Additionally, no signs of immune rejection of the endothelial grafts were observed. By contrast, the corneas of the rabbit control group, which had only the Descemet membrane peeled off, did not recover and exhibited oedema throughout the 6-week follow-up period. The same results were also observed in the rabbit group that received the silk fibroin transplant without cultured corneal endothelial cells. This study (20) showed that silk fibroin can be an effective artificial Descemet membrane for the ex vivo culture of corneal endothelial cells in preparation for a silk fibroin endothelial graft.

### Silk for Ocular Tissue Engineering: Retinal Bruch’s Membrane

One of the approaches for treating retinal degeneration caused by diseases such as age-related macular degeneration involves the transplantation of a monolayer of retinal pigment epithelial cells (88,89). Exciting advances are being made in this area; for example, Phase I clinical trials have shown successful transplantation of human embryonic stem cell-derived retinal pigment epithelium using porous fibronectin-coated polyester membranes (90). These non-biodegradable membranes were derived from cell culture Transwells®. However, one of the drawbacks of this type of procedure is the potential for folding of the retinal pigment epithelial cell sheet after transplantation and the persistence of the membrane (89,91). The use of silk fibroin as a substrate for *in vitro* proliferation of retinal pigment epithelial cells can potentially replace the Bruch’s membrane after transplantation.

In the reconstruction of the retina, the material will be located close to neurological tissue, so biocompatibility of a thin, mechanically strong support is a key requirement. Shadworth and colleagues achieved silk fibroin beta-sheet formation by water annealing at room temperature prior to seeding with cells (19). The cells on the 3 μm thick silk fibroin film exhibited characteristics of matured retinal pigment epithelial cells, such as cobblestone morphology accompanied by pigmentation, within a 12-week timeframe (19). Over this time, the retinal pigment cells proliferated and slowly established dense surface microvillous structures (19). The matured retinal pigment epithelium cells also developed a capacity to perform phagocytosis as well as to secrete essential growth factors, such as pigment epithelium-derived factor and vascular endothelial growth factor. Although this study did not evaluate the *in vivo* implantation of the synthetic Bruch’s membrane, the reported data indicate the potential for such an approach for the treatment of retinal degeneration diseases.

### Silk for Ocular Wound Healing

The regenerative properties of silk fibroin in ocular tissue engineering can be extended to other ocular treatments, such as wound healing of the ocular surface. The ocular wound healing process typically begins by the migration of epithelial cells to the wound area, followed by the proliferation and differentiation of the limbal stem cells to replace the damaged tissue (23). However, in contrast to its action during tissue engineering, silk fibroin does not act as a substrate for the cells to differentiate and proliferate during the wound healing process, rather, it has a pharmacological effect to direct the adjacent healthy epithelial cells towards the wounded area to start the healing process.

The migration, proliferation and adhesion of the epithelial cells to a wound area have been demonstrated *in vitro* using limbal epithelial cells and a scratch wound assay (23). To initiate the scratch wound assay, a healthy epithelial cell layer was generated from limbal epithelial cells. A wounded region was generated by scratching the cell monolayer, followed by washing and the addition of fresh growth media with different concentrations (0.2, 0.4 and 0.5%*w*/*v*) of a proprietary solution of silk-derived protein (Silk Technologies, Ltd. Plymouth, Minnesota) with PBS as a vehicle control. Cell migration and proliferation were monitored by time-lapse imaging. To differentiate between migration and proliferation, the mitotic inhibitor hydroxyurea was used to suppress cell growth. Cell migration rate was highest at 0.4% concentration, followed by 0.2% and then the PBS vehicle control. However, cell migration was slower with 0.5% silk-derived protein than with PBS and significantly slower than with 0.2% silk-derived protein. Cell proliferation significantly increased in the presence of silk-derived protein relative to control cells. Cell adhesion also significantly increased for all silk-derived protein concentrations when compared to the PBS control.

**Fig. 2:**
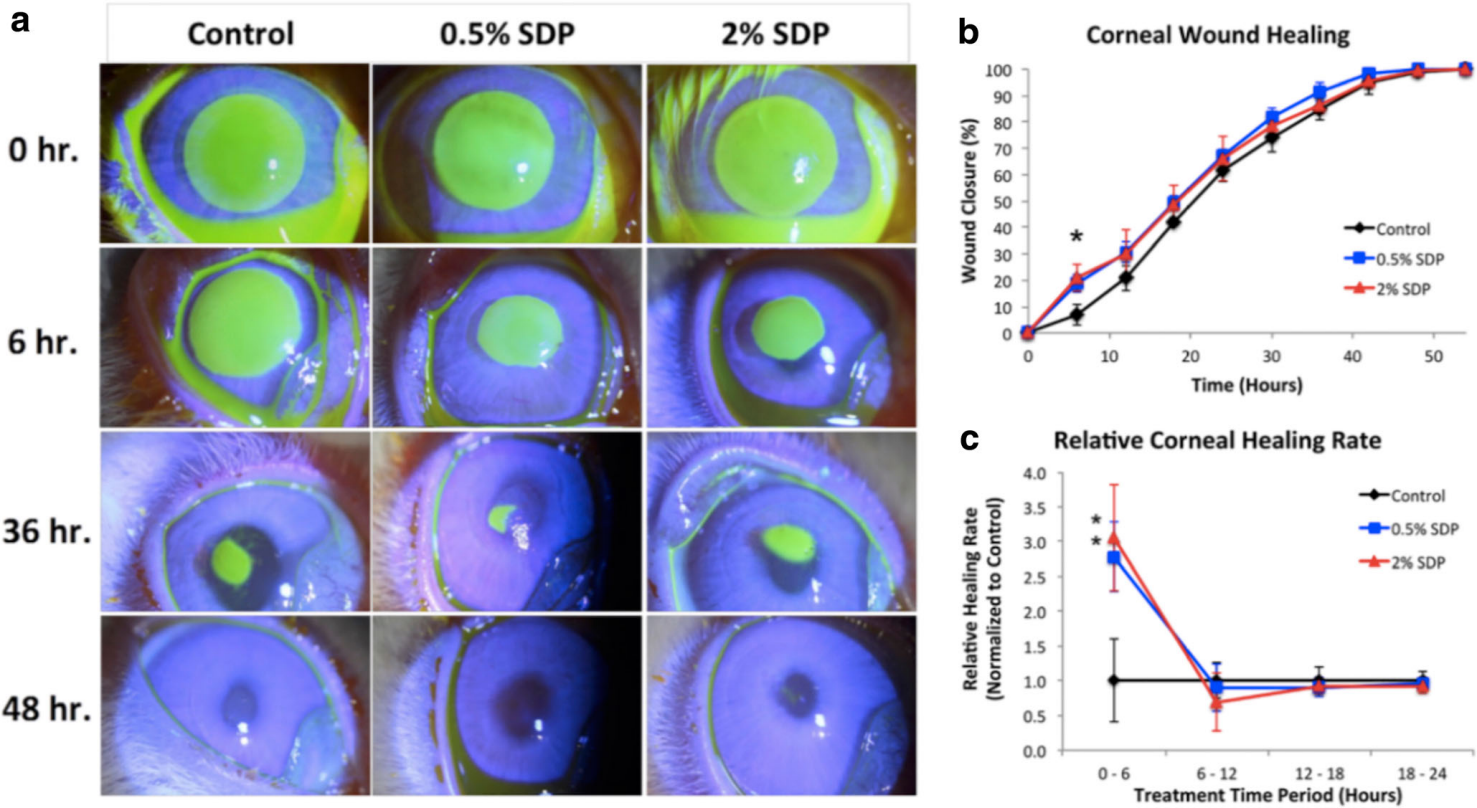
Impact of silk fibroin-derived protein on the healing of a rabbit corneal wound. (**a**) Fluorescein signal (i.e. wound area) at indicated time points of rabbit corneas subjected to epithelial debridement. (**b**) Corneal wound healing and (**c**) normalised healing rates between 6 and 24 h post-debridement. Reproduced with permission from (24).

The wound healing effect of silk-derived protein was then assessed in a rabbit corneal injury model (24) (Fig. 2). The rabbit corneas were denuded of their epithelial layer and then treated with silk-derived protein at 0.5% and 2.0% concentration as an eye drop formulation; PBS eye drops served as the control. Across all treatment groups, the rabbits exhibited >95% corneal surface repair within the first 48 h post treatment, although the rate of repair was highest following treatment with the silk-derived protein. The authors chose to evaluate the 0.5% and 2.0% concentrations, even though the 0.5% concentration performed worst in an *in vitro* cell migration study (23).

The wound healing effect of silk fibroin has been postulated to have a therapeutic effect on the dry eye disease. This disease is marked by a destabilisation of the tear film, which leads to lower tear production and, ultimately, damage to the corneal epithelial cells and loss of the conjunctival goblet cells (25). The authors (25) used a dry eye mouse model to evaluate the impact of silk fibroin on tear production, corneal irregularity score, corneal epithelial cell detachment, density of conjunctival goblet cells and inflammatory factors in the lacrimal gland. Dry eye conditions were simulated in 12-week-old mice by exposing them to 30–40% ambient humidity and injecting them with scopolamine hydrobromide for 10 days. The study consisted of two test groups (treated with either 1 or 5 mg/ml silk fibroin solution) and one control group (treated with PBS). All formulations were delivered in an eye drop format using an aqueous silk fibroin solution derived from *B. mori* cocoons.

The dry eye model group (desiccation stress treatment group) showed a 76% reduction in tear production, with a 16-fold increase in epithelial cell detachment, and a 56% decrease in goblet cell density, when compared to the healthy control group that was not subjected to stress. After a 10-day treatment with either PBS or 1 or 5 mg/ml silk fibroin, all groups showed improved tear production: the PBS group showed 3-fold increase, while both silk fibroin-treated groups showed a 4-fold increase.

Corneal irregularity returned to baseline after 10 days in all 3 groups, with the fastest rate observed for the silk fibroin groups. Epithelial cell detachment showed a 94% decrease in the silk fibroin groups *versus* a 31% decrease in the PBS group. The conjunctival goblet cell densities also increased after treatment with PBS or silk fibroin, but the densities were greater for the silk fibroin groups than for the PBS group. The amounts of inflammatory factors (e.g. TNF-α, MMP-2, MMP-9, ICAM-1, and VCAM-1) were statistically lower in the silk fibroin treatment groups than in the PBS group or the control (no treatment) group. The authors concluded that silk fibroin has the potential to increase tear production in dry eye disease through an increase in the numbers of conjunctival goblet cells and an anti-inflammatory effect of silk fibroin in the lacrimal gland (25).

### Silk for Ocular Drug Delivery

The ability of silk fibroin to self-assemble into hydrogels in an aqueous solution, coupled with its biocompatibility, makes it an attractive polymer for ocular drug delivery of macromolecules, such as antibodies and other therapeutic proteins (reviewed in (60)). One example is the incorporation of bevacizumab, an antibody that inhibits the protein vascular endothelial growth factor for the treatment of wet age-related macular degeneration, into a silk hydrogel. Delivery of this hydrogel into the vitreous of Dutch belted rabbits via intravitreal injections (27) revealed the potential of silk hydrogels for ocular drug delivery, including the capacity for aseptic processing, biocompatibility and sustained-release capability (27).

Silk fibroin can be assembled into a hydrogel format in aqueous solutions; therefore, concentrated sterile solutions of silk fibroin and bevacizumab can be mixed together, followed by sonication-induced gelation. This process can be performed aseptically and the resulting test article formulations are sterile and endotoxin-free in accordance with United States Pharmacopeia guidelines. After injection into the rabbit vitreous, no differences were noted in the occurrence of adverse events between the rabbits treated with bevacizumab-loaded hydrogels and the controls, thereby substantiating the authors’ claim of biocompatibility. Furthermore, the bevacizumab incorporated into the silk hydrogel showed a sustained release for 90 days, in contrast to the commercial solution formulation of bevacizumab, which showed discontinuous release after 30 days.

Lovett and colleagues demonstrated the promising potential of silk hydrogel as a vitreous drug delivery system (27), although the need to develop second generation silk hydrogels appeared likely. First, the silk hydrogel was not completely transparent in the vitreous, thereby risking the obscuring of vision. Second, the capacity for silk hydrogel biodegradation in the vitreous is currently unknown, and the reported observations are inconclusive because a volume reduction in the silk hydrogel could occur due to shrinkage rather than biodegradation. The *in vitro* bevacizumab release profile was better from the silk hydrogel than from the control (at >33 days), but the achieved concentration of 500 ng/mL (or less) was below the therapeutic level required for biological efficacy, based on published and calculated pharmacokinetics data (92–95). The *in vivo* data were also below the therapeutic window. Based on the *in vitro* data, the total recovery of bevacizumab was apparently incomplete.

The total recovery of an encapsulated protein can be affected by many factors related to the interactions of the protein with the silk fibroin matrix. For instance, Guziewicz and colleagues found that the cumulative release of a murine IgG1 monoclonal antibody, which is synonymous with its total recovery, decreased incrementally with increasing silk fibroin concentrations in a lyogel formulation (96). Lyogels are lyophilised formulations of self-assembled hydrogels. The increase in silk fibroin concentration directly impacted the density of the lyogel, which controls the solvent penetration and disruption of the silk-antibody hydrophobic interactions. These hydrophobic interactions could be countered by pH effects and additional, secondary ionic interactions, which, although weaker, could improve the release and recovery of the antibody. This study also showed that the silk matrix can adversely affect an encapsulated antibody containing important methionine residues, as oxidation of these residues increased with increasing silk density. Oxidation increased from 6% to 28% with a 3% silk lyogel and to 34% with a 7% lyogel (96). Although the observed oxidation in this study did not affect the biological activity of the model protein, such chemical modification has been implicated in adverse effect to other protein therapeutics and applications (97–99).

In addition to the solution pH, different types of salts and the ionic strength of a solution can significantly affect the ionic interactions between a protein drug and its surrounding silk fibroin matrix (100). Chaotropic salts can disrupt these ionic interactions, as shown by the inclusion of sodium thiocyanate, which improved the release of the protein drug. Conversely, kosmotropic salts, such as sodium sulphate, can entropically drive the complex coacervation between proteins with opposing net charges, such as protamine and silk fibroin at neutral pH.

The ability of silk fibroin to interact ionically with an oppositely charged molecule can be exploited for topical drug delivery on the eye surface. For example, Dong and colleagues utilised the electrostatic interactions between the soluble silk fibroin and liposomes to develop a formulation for topical delivery (26). Their objective was to extend the residence time of ibuprofen on the eye surface by encapsulating it into liposomes coated with silk fibroin. In this application, the silk fibroin served as a potential mucoadhesive biopolymer to retain the drug on the eye surface to ensure sustained delivery.

The silk fibroin was prepared by degumming *B. mori* silk cocoons twice for 20 min in sodium carbonate. After washing and drying, the collected silk fibroin was dissolved in a mixture of calcium chloride, ethanol and water at a molar ratio of 1:2:8 while stirring at 78°C for 2, 4, 6, and 8 h. The differences in dissolution time yielded different fractions of silk fibroin with different molecular weights. After water dialysis, the resulting silk fibroin solution was lyophilised for 48 h and then reconstituted to 5% *w*/*v* with PBS for preparation of silk fibroin coated liposomes. The liposomes containing ibuprofen were coated with silk fibroin by adding a silk fibroin solution to a final concentration of 0.5%, 1.0% or 2.0% (*w*/*v*). After 1 h of gentle agitation, methanol was added to induce the structural transition of the silk fibroin and to precipitate the silk fibroin coated liposomes for collection and washing.

Gel electrophoresis showed that the molecular weights of the silk fibroin protein chains decreased as the dissolving time increased. Fourier-transform infrared spectroscopy and X-ray diffraction data confirmed beta-sheet formation. *In vitro* corneal permeation experiments showed that ibuprofen release was the slowest from silk-coated liposomes, when compared to uncoated liposomes and an aqueous formulation of ibuprofen. While the apparent permeability coefficients, derived from the transport flux of ibuprofen through the thickness of the corneal membrane, were the same for all tested formulations, a sustained permeation was observed for the silk-coated liposome formulation (26). The data suggest that silk fibroin-coated liposomes could be a potential drug delivery system for ophthalmic formulations.

### Silk for Ocular Prostheses

The transparent property of silk fibroin renders this biopolymer an interesting candidate for use as a corneal prosthesis to improve visual acuity. Applegate and colleagues generated elastic silk hydrogels that were chemically crosslinked using riboflavin (21), a photoinitiator that has been used to crosslink corneal collagen for treatment of corneal ectatic diseases (101). These authors examined the mechanisms of silk fibroin crosslinking by riboflavin and found that the elastic crosslinked silk hydrogels adhered to the corneal collagen of enucleated and de-epithelialised porcine eyes. The silk hydrogels maintained their 40 μm thickness and adhered to the surface of the eyes even after extensive washing with PBS. By contrast, the silk films without riboflavin and silk films containing riboflavin but not illuminated were both readily washed from the eye surface. The authors then conducted an *in vitro* assessment of the photocrosslinking process using heavy water D_2_O, sodium azide and superoxide dismutase and determined that the silk gelation was driven principally by dityrosine formation radicalised by the excited riboflavin. The formation of dityrosine complexes from the tyrosine amino acid residues covalently bound the silk molecules together to form the elastic hydrogel (21) (as opposed to physically cross-linked silk hydrogels, which are brittle (60)).

**Fig. 3:**
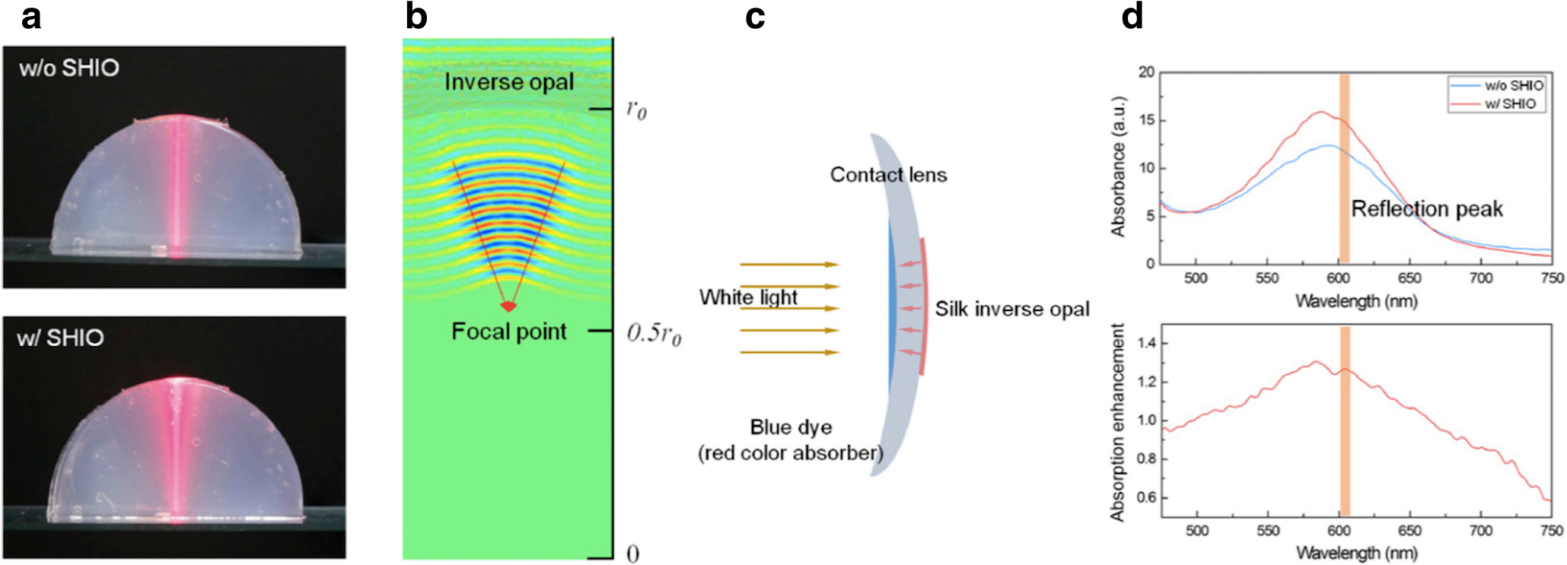
Deformable and conformable silk fibroin hydrogel three dimensional photonic crystal (i.e. opal). (**a**) Photographic images showing the reflection behaviour of a red laser beam in the absence and presence of an opal conformally placed on an agarose gel hemisphere (an artificial eye model). (**b**) The in silico simulation reveals that the conformal opal acts as a concave mirror, focusing the reflected light. (**c**) and (**d**) Schematic diagram and experiment to prove better absorption of incident light by opal. Copyright (2017) National Academy of Sciences, reproduced with permission from (22).

Crosslinked silk hydrogels can be engineered to mimic nature’s photonic crystals. Min and colleagues evaluated a silk-based crosslinked hydrogel as an ocular prosthesis for high-technology applications that include night vision, infrared vision and *in vivo* biosensors (22) (Fig. 3). The synthetic photonic crystals were generated by stacking layers of 300 nm poly(methyl methacrylate) spheres on a silicon substrate, followed by photocrosslinking of a silk fibroin and stilbene solution, which was poured into the layers of the sphere template. The silicone substrate was then physically removed and the spheres were washed away with acetone. The resulting synthetic silk-based optical nanostructure was a deformable and conformable silk hydrogel inverse opal (i.e. 3 dimensional photonic crystal) that displayed an opalescent property under white-light illumination in different media, such as air, water and isopropanol.

The optical properties of the silk hydrogel inverse opal were the direct effects of the transparent silk fibroin coupled with the pseudophotonic band gap associated with the lattice geometry and the reflective index of the media. Min and colleagues showed that the pseudophotonic band gap, corresponding to the exhibited structural colour, can be manipulated using mechanical forces, such as stretching and pressure, to alter the interplanar spacing. For instance, a stretching strain of 10% induced a 7 nm blue shift of the reflectance λ_peak_, while an increase to 20 kPa resulted in an approximately 4 nm blue shift. Using an eye model consisting of hemispheres of agarose gel and soft contact lenses, the authors showed that the silk hydrogel inverse opal enhanced the absorption of incident white light in the broad range of 510–660 nm at a maximum factor of approximately 1.3 (Fig. 3). The observed optical properties of the silk hydrogel inverse opal showed the potential for engineered silk hydrogel to serve as an artificial *tapetum lucidum,* which also can enhance the incident light available to the photoreceptors to provide superior night vision (emulating nocturnal animals) and as a glaucoma indicator through the detection of intraocular pressure (22).

### Silk and the Advanced, Electronically Enhanced Ocular Drug Delivery Concepts

Silk fibroin is an interesting example of a biological liquid crystal and it displays long-range partial orientation of molecular motifs. In particular, the molecules are constrained into parallel planes (smectic A phase symmetry), which imparts structural strength and flexoelectric properties, where application of strain generates polarisation in a manner similar to but distinct from piezoelectricity. Helicoid motifs are common in biological systems, such as silk, DNA and cellulose (102) and show layer structures and twists. Application of strain allows stretching and recovery of silk fibroin and increasing concentrations of solutions promote tangling that leads to increases in viscosity before the emergence of crystallinity. These interesting properties bridge the gap between structural properties and those useful in fabricating devices that respond to changes in, for example, transparency or electrical polarisation.

Silk has many potential roles as a material in ocular applications as it can be processed into a variety of forms including weavable fibres, as well as films, membranes, particulates and hydrogels which can be loaded with drugs and made into responsive systems that would lead to better control of delivery rates. These systems would fit into the category of highly biocompatible physical engines capable of sensing and controlling flux. Koh and colleagues specifically reviewed this need as ‘a ‘green’ approach for manufacturing next-generation wearable, implantable, self-resorbable electronics and energy-devices’ (103). This approach requires natural materials or adapted natural materials that, in this context, can outperform synthetic materials. Nanotechnology approaches to modify cast films (e.g. nano-patterning or nano-sculpturing) must be complemented with organic semiconductors and energy sources. Koh and colleagues reviewed the use of high throughput hot- or room-temperature nanoimprinting techniques to create sub-100 nm features on silk fibroin (103). Subsequent treatment of the film with methanol then causes crystallisation (converting soluble silk I to water insoluble silk II) and increases the glass transition temperature. This approach can then be used to build photonic devices, such as diffractive systems, holograms prisms and microlens arrays (4).

Field effect transistors have extremely high impedance, so tiny currents can be used to gate a switch (i.e. a transistor). Organic field-effect transistors were described in 1987, although they were largely hybrid inorganic-organic devices. A polymer has advantages over doped silicon for electric switching and amplification in terms of flexibility and the ability to be the construction material for machines using printing and spin-coating techniques (104). Silk fibroin can be used as the dielectric and sandwiched between the gate and conducting layer, thereby replacing silicon dioxide. Silk fibroin has been used in the fabrication of diodes, including those which are light-emitting, allowing transmittance of information though light channels and films (4). Silk fibroin has also been exploited in the fabrication of light-sensing or wirelessly powered devices that allows remote triggering of drug release and monitoring of events deep within body tissues (105), for example the eye.

Silk fibroin based “soft robots” or devices (106) are intriguing and have the potential to contribute to advanced healthcare applications. However, the robotisation of machines typically requires a memory system containing the instruction set. This type of system is generally associated with some kind of chip, and the chip materials usually far outlast the use of the device. Bioresorbable electronics based on mono crystalline silicon have now been described, while organic substrates as diverse as rice paper, cheese, charcoal, seaweed and silk fibroin have been reported (reviewed in (107)). For example, Song and colleagues consider the use of zinc oxide, magnesium and silk firboin to construct resistive random access memory devices, which, at the end of life, would completely dissolve and be resorbed (108). Resistive random access memory devices have very low operational voltages and low power requirements, extremely fast write read speeds, and are non-volatile and reliable. Carbon-based resistive random access memory devices are one of the most promising candidates as these use oxygen-based redox reactions in switching (109). Gorgula and colleagues described a transparent flexible device constructed from gold nanoparticles embedded in a silk fibroin matrix with an ON/OFF ratio of resistance of more than 6 orders of magnitude (110). This was achieved by filamentary switching, a process that involves alteration of the molecular dimensions of a structure, such as a macromolecule or polymer. Reputedly, this kind of solid-state device can model the biological synapse (111).

Unlike silk fibroin, sericin is generally not sufficiently strong or elastic to make membranes from pure material; however, it will adhere onto other matrices as a thin film. For example, Zhang (43) quotes a Japanese patent describing the production of a distortion-free liquid crystal display using sericin. Cross-linking the protein improves its physical stability and the treated protein can be used to make hydrophilic membranes.

Electric textiles have also been fabricated from a weft yarn of copper wire wound onto silk fibroin, or carbonised silk (112), to make wearable electronics. Silk fibroin has been considered for the fabrication of thin, flexible and lightweight devices for wearable and implantable systems (4,107). Microfluidic silk fibroin devices with binary fluid channels have been produced using sacrificial gelatine moulding (113). Three-dimensional printing within nanoclay:silk fibroin gel ‘free-form printing’ allows complex microstructured objects, including silk-based cantilevers, to emerge from a submerged granular hydrogel. The authors propose that these cantilevers could be controlled through signals sent through silk-based bio-inks (114). Here, the issue of the electrical resistance must be considered. Koh and colleagues reviewed the technologies to reduce ‘haze’, the low transparency due to scatter, which can be improved to values associated with glass by reducing the cross-sectional area of the fibre and by using metal-doped silks (103). The utility of silk, with its inherent biocompatibility and comfort, has attracted the imagination of the electronics engineering industry. For example, plasticised silk electrodes that conform to the skin and are highly stretchable (115). These materials could be used in the fabrication of scleral devices, including supports for sensors, reservoirs and directing channels.

## Conclusions

While silk has been used as a suture material for millennia, the advent of recombinant silks and our ability to regenerate the silk fibroin has opened up numerous applications for silk in the treatment of eye disorders. The use of the silk biopolymer for tissue engineering applications has been pursued for several decades, whereas its use in drug delivery applications has emerged over the past 10 years. The use of silk fibroin as a biological active polymer is now gaining momentum. As we continue to unravel the secrets of silk, we can expect to see more silk-inspired materials to enter clinical assessment and eventual clinical use.

## ABBREVIATIONS

PBS: Phosphate buffered saline
PEG: Polyethylene glycol
RGD: Arginine-glycine-aspartic acid
